# Determination of (4*E*,6*E*,12*E*)-Tetradecatriene-8,10-diyne-1,3-diyl Diacetate in Rat Plasma and Tissues by HPLC-UV Method and Their Application to a Pharmacokinetic and Tissue Distribution Study

**DOI:** 10.1155/2014/249061

**Published:** 2014-02-24

**Authors:** Yan Huo, Yu-Qiang Liu, Zhong-Xu Bai, Qian Cai

**Affiliations:** School of Medicine, Liaoning University of Traditional Chinese Medicine, Dalian 116600, China

## Abstract

In China *Atractylodis Rhizoma* is widely used for the treatment of rheumatic diseases and digestive disorders. Stir-frying with wheat bran is the most common processing method. In order to clarify the influence of processing on pharmacological properties of* Atractylodis Rhizoma*, an investigation was carried out to compare the pharmacokinetics and tissue distribution of typical constituent after oral administration of raw *Atractylodis Rhizoma* and processed ones. A simple, rapid, and sensitive high performance liquid chromatography with UV detection was developed and validated for the determination of (4*E*,6*E*,12*E*)-tetradecatriene-8,10-diyne-1,3-diyl diacetate in rat plasma. A chromatography was carried out on Diamonsil C_18_ (250 × 4.6 mm; 5 **μ**m) analytical column, using a mobile phase which consisted of acetonitrile and 0.1% phosphoric acid water (60 : 40, v/v) at a flow rate of 1.0 mL*·*min^−1^. The wavelength was set at 336 nm. The LLOQ of (4*E*,6*E*,12*E*)-tetradecatriene-8,10-diyne-1,3-diyl diacetate was 0.00143 ***μ***g*·*mL^−1^. Both accuracy and precision were satisfactory. The pharmacokinetic results showed that the *T*_max⁡_ was 1 hour in advance and the *C*_max⁡_ was increased after processing. Tissue distribution showed that the highest level was in spleen. And the concentrations in the spleen were increased after stir-frying with bran.

## 1. Introduction

In traditional Chinese medicine, *Atractylodis Rhizoma *is the dried root and stems from *Atractylodes lancea* (Thunb.) DC. or *Atractylodes chinensis* (DC.) Koidz. The medicinal herb is widely known as Cangzhu in China. And it is widely used for the treatment of rheumatic diseases, digestive disorders, mild diarrhea, and influenza [1]. In clinic, *Atractylodis Rhizoma* is often processed by stir-frying with wheat bran with the aim of reducing its dryness and increasing the function of tonifying spleen [2, 3].

*Atractylodis Rhizoma* is rich of essential oil including sesquiterpenes and polyethylene alkynes, which are the main active components in this medicine [4]. Recent researches have shown that polyethylene alkynes exhibit various desirable pharmacological effects including anti-inflammatory, antibacterial, and antiarrhythmic activity [5]. (4*E*,6*E*,12*E*)-tetradecatriene-8,10-diyne-1,3-diyl diacetate is one of polyethylene alkyne components. Some literature reported HPLC method for determination its content in *Atractylodis Rhiomzoma *[6–8].

However, there have been few methods available for its quantification in biosamples and few reports on its pharmacokinetic study and tissues distribution until now. The *in vivo* study of (4*E*,6*E*,12*E*)-tetradecatriene-8,10-diyne-1,3-diyl diacetate, an active component of* Atractylodis Rhizoma*, would be necessary and helpful for further clinical application and explanation of the processing mechanism. The present paper developed a new and simple RP-HP LC method for quantification of (4*E*,6*E*,12*E*)-tetradecatriene-8,10-diyne-1,3-diyl diacetate in rat plasma and tissues after oral administration of raw and processed *Atractylodis Rhizoma, *respectively. This fully validated method was successfully applied to a pharmacokinetic and tissue distribution study of (4*E*,6*E*,12*E*)-tetradecatriene-8,10-diyne-1,3-diyl diacetate in rats for the first time.

## 2. Experimental

### 2.1. Chemicals and Reagents

**Figure 1:**
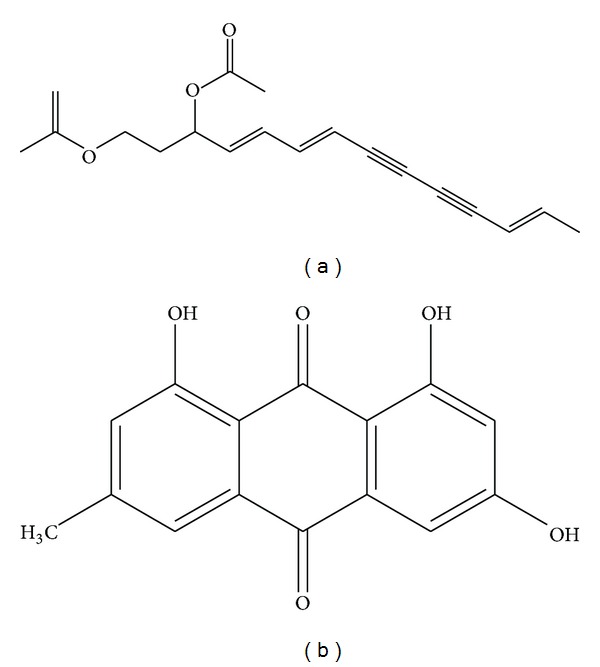
Chemical structures of (4*E*,6*E*,12*E*)-tetradecatriene-8,10-diyne-1,3-diyl diacetate (a) and Emodin (b).

The (4*E*,6*E*,12*E*)-tetradecatriene-8,10-diyne-1,3-diyl diacetate (purity, 98%) was supplied by Traditional Chinese Medicine Standardization Research Center (Shanghai, China). The IS called Emodin (purity, 98%) was supplied by the National Institute for Food and Drug Control (Beijing, China). The chemical structures of (4*E*,6*E*,12*E*)-tetradecatriene-8,10-diyne-1,3-diyl diacetate and IS are shown in Figure 1.

HPLC grade acetonitrile was purchased from Fisher Scientific Company (New Jersey, USA) and Pure water was supplied by Wahaha Company (Hangzhou, China). Analytical grade ethanol and chloroform were from Baierdi Company (Beijing, China). *Atractylodis Rhizoma *was identified by Professor Li Feng (*Liaoning University of TCM)* according to the standards of Chinese Pharmacopoeia 2010. The processed *Atractylodis Rhizoma* comes from the same batch *Atractylodis Rhizoma*. The herb was stored in a cool and dry place.

### 2.2. Preparation of *Atractylodis Rhizoma *Solution

*Atractylodis Rhizoma* (50 g) was crushed into powder and soaked into 600 mL of 80% ethanol for 24 h and then percolated at 2 mL·min^−1^, and Ethanol was evaporated to near dryness under reduced pressure to get the residue. Distilled water was added into the residue and then vortexed for 10 min. The final concentration of *Atractylodis Rhizoma* solution was 2 g·mL^−1^ [9]. The sample was stored in dry and dark place before use.

### 2.3. Apparatus and HPLC Conditions

The liquid chromatographic system consisted of an LC-10 AD pump (Shimadzu, Kyoto, Japan) with a 20 *μ*L loop (Cotata, CA, USA) and an SPD-10A ultraviolet-visile detector (Shimadzu, Kyoto, Japan). A LC-10 AD workstation was used for data acquisition. A Diamonsil C_18_ analytical column (250 × 4.6 mm; 5 *μ*m) from Dikma Technologies (Dalian, China) was used. The mobile phase consisted of acetonitrile and 0.1% phosphoric acid water (60 : 40, v/v) at a flow rate of 1 mL·min^−1^. The detection wavelength was set at 336 nm. All the measurements were performed at room temperature, and the injection volume was 20 *μ*L.

### 2.4. Preparation of Standard Solution and Quality Control Samples

Stock solutions of (4*E*,6*E*,12*E*)-tetradecatriene-8,10-diyne-1,3-diyl diacetate and IS with concentrations of 0.143 mg·mL^−1^ and 0.0504 mg·mL^−1^, respectively, were prepared in methanol and stored at −20°C and dark place until use. When we used the standard solution, the working concentration of (4*E*,6*E*,12*E*)-tetradecatriene-8,10-diyne-1,3-diyl diacetate and IS were 286 *μ*g·L^−1^ and 5.04 *μ*g·L^−1^, respectively. Calibration standards of (4*E*,6*E*,12*E*)-tetradecatriene-8,10-diyne-1,3-diyl diacetate were prepared by spiking the appropriate amount of the working solutions into 200 *μ*L blank rat plasma or tissue homogenates. To plasma samples, the final concentrations of calibration standard samples were 0.003575, 0.00715, 0.0143, 0.03575, 0.0715, 0.143, and 0.2145 *μ*g·mL^−1^. Quality control (QC) samples were prepared at low, medium, and high concentrations of 0.00375, 0.03575, and 0.0715 *μ*g·mL^−1^. To tissue homogenates, the final concentrations of calibration standard samples were 0.00143, 0.003575, 0.00715, 0.0143, 0.0286, 0.03575, and 0.0715 *μ*g·mL^−1^. Quality control (QC) samples were prepared at low, medium, and high concentrations of 0.00375, 0.03575, and 0.0715 *μ*g·mL^−1^ for tissue homogenates.

### 2.5. Sample Preparations

For plasma samples, the 200 *μ*L of rat plasma was mixed with 20 *μ*L IS (0.00504 mg·mL^−1^). After protein was precipitated with 1,000 *μ*L of acetonitrile in 1.5 mL polypropylene tube by vortexing for 2 min, the sample was centrifuged at 10,000 rpm·min^−1^ for 5 min. The supernatant was transferred into a 5.0 mL tube and added with 1,000 *μ*L of chloroform, extract and the under organic phase was transferred to another tube and evaporated to dryness at 40°C with nitrogen. The resulting extract was dissolved in 50 *μ*L of methanol, and vortex mixed for 2 min. After centrifugation at 10,000 rpm·min^−1^ for 5 min, 20 *μ*L supernatant was injected for analysis [10–12]. For tissue homogenate, each weighed tissue sample was thawed and the homogenized in ice-cold physiological saline (2 mL). Then a 200 *μ*L of tissue homogenate was taken and processed further like the plasma samples.

### 2.6. Method Validation

#### 2.6.1. Specificity

The selectivity of the method was demonstrated by comparing chromatograms of blank plasma samples and tissue homogenate (without IS) obtained from rats and plasma samples and tissue homogenate spiked with the analytes and IS and plasma samples and tissue homogenate after an oral dose. All blank plasma samples and tissue homogenates were prepared and analyzed to ensure the absence of interfering peaks.

#### 2.6.2. Calibration Procedure

The linearity of the method was assessed by plotting calibration curves in plasma at seven concentration levels in triplicate on three consecutive days. The lower limit of quantification (LLOQ) was defined as the lowest concentration of the calibration curve that was measured with accuracy and precision by analyzing samples in six replicates at the concentration of 0.00143 *μ*g·mL^−1^ for (4*E*,6*E*,12*E*)-tetradecatriene-8,10-diyne-1,3-diyl diacetate.

#### 2.6.3. Accuracy and Precision

Intraday precision and accuracy were evaluated by analysis of the three QC samples with six determinations per concentration at the same day, whilst the interday precision and accuracy were measured over three consecutive days. The precision was defined as the relative standard deviation (RSD%), while accuracy was determined by calculating the percentage deviation observed in the analysis of QC samples and expressed by relative error (RE%). The accepted criteria for the data were that the precision and accuracy should not exceed 15%, except at the LLOQ, where it should not exceed 20%.

#### 2.6.4. Extraction Recovery and Stability

The extraction recoveries of (4*E*,6*E*,12*E*)-tetradecatriene-8,10-diyne-1,3-diyl diacetate were determined at low, medium, and high level of QC samples. Recoveries were calculated by comparing the observed peak area ratios in biosamples to those nonprocessed standard solutions at the same concentrations. The recovery of IS was determined in the same way at the concentration of 0.00504 mg·mL^−1^.

The stability of (4*E*,6*E*,12*E*)-tetradecatriene-8,10-diyne-1,3-diyl diacetate in plasma and tissue was determined under different storage or handling conditions. Short-term stability was assessed by analyzing QC samples kept at room temperature for 8 h. Freeze-thaw stability was evaluated at three consecutive freeze-thaw cycles. Long-term stability was studied by assaying samples following a period of 10 days of storage at −20°C.

### 2.7. Applications in Pharmacokinetic Studies

All the studies on animals were in accordance with the Guidelines for the Care and Use of Laboratory Animals. Healthy Sprague-Dawley rats (250 ± 20 g) were purchased from The Medical University of Dalian (Dalian, China) and acclimated in the laboratory for one week before to the experiments. Rats for oral ingestion were fasted for 12 h with free access to water. Rats were oral administration raw and processed *Atractylodis Rhizoma* at a single dose of 40 g·kg^−1^, respectively.

For plasma samples, twelve rats were randomly assigned to two groups for pharmacokinetic investigation (*n* = 6 per group). The blood sample (0.5 mL) was collected at 0, 0.17, 0.33, 0.5, 1, 2, 3, 4, 6, 8, 12, and 24 h. All samples were immediately transferred into heparinized tubes and centrifuged for 5 min at 10,000 rpm·min^−1^. The supernatant was stored at −20°C and dark place until use.

### 2.8. Tissue Distribution Study

For tissue distribution study, forty-eight rats were divided into eight groups (*n* = 6 per group) randomly.

After oral administration four groups rats raw *Atractylodis Rhizoma* at a single dose of 40 g·kg^−1^ and the other four groups rats processed *Atractylodis Rhizoma* at a single dose of 40 g·kg^−1^. Heart, liver, spleen, lung, kidney, stomach, small intestine, and large intestine were collected at 0.5, 2, 4, and 8 h. Tissue samples were weighed 0.1 g rapidly, rinsed with physiological saline to remove the blood or content, blotted on filter paper, and then stored at −20°C and dark place until use.

### 2.9. Statistical Analysis

HPLC analysis procedure was applied to analyze plasma concentration-time profiles of (4*E*,6*E*,12*E*)-tetradecatriene-8,10-diyne-1,3-diyl diacetate. Data was processed by noncompartmental method using Drug and Statistics (DAS) 2.0 software package (Chinese Pharmacological Society, Shanghai, China).

## 3. Results and Discussion

### 3.1. Method Validation

#### 3.1.1. Specificity

**Figure 2:**
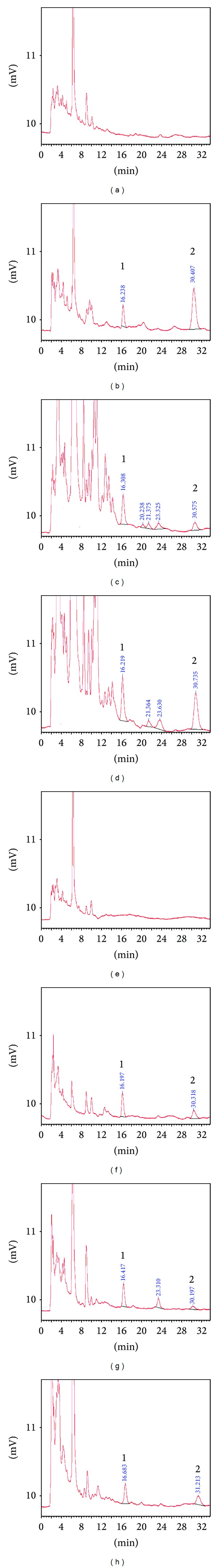
Chromatograms of blank plasma (a); blank plasma spiked with (4*E*,6*E*,12*E*)-tetradecatriene-8,10-diyne-1,3-diyl diacetate 20 *μ*L (0.143 *μ*g·mL^−1^) and IS 20 *μ*L (0.00504 mg·mL^−1^) (b); rat plasma sample (4 h) after oral administration of raw *Atractylodis Rhizoma *40 g·kg^−1^ (c); rat plasma sample (4 h) after oral administration of processed *Atractylodis Rhizoma* 40 g·kg^−1^ (d); Chromatograms of blank tissue homogenate (e); blank tissue homogenate with (4*E*,6*E*,12*E*)-tetradecatriene-8,10-diyne-1,3-diyl diacetate 20 *μ*L (0.286 *μ*g·mL^−1^) and IS 20 *μ*L (0.00504 mg·mL^−1^) (f); spleen sample (2 h) after oral administration of raw *Atractylodis Rhizoma* 40 g·kg^−1^ (g); spleen sample (2 h) after oral administration of processed *Atractylodis Rhizoma* 40 g·kg^−1^ (h).

The representative chromatograms for determination of (4*E*,6*E*,12*E*)-tetradecatriene-8,10-diyne-1,3-diyl diacetate in plasma and tissues are shown in Figure 2. The retention time of Emodin (IS) was about 16.30 min and (4*E*,6*E*,12*E*)-tetradecatriene-8,10-diyne-1,3-diyl diacetate was about 30.45 min. It was indicated that analytes and IS were well separated and no interferences were detected from endogenous substances or metabolites.

#### 3.1.2. Linearity of Calibration Curve and Lower Limit of Quantification

The calibration curves were linear over the concentration range of 0.003575–0.2145 *μ*g·mL^−1^ in rat plasma and 0.00143–0.0715 *μ*g·mL^−1^ in tissue homogenates by weighted (1/*x* ^2^) linear least-squares regression method. The correlation coefficient values of the calibration curves were over 0.9900. The RE of the back-calculated values of the standards from their nominal values were constantly within 15% for all values, including the LLOQ. The LLOQ measurement showed the respective averages 0.003575 *μ*g·mL^−1^ with RSD 7.68% for rat plasma and 0.00143 *μ*g·mL^−1^ with RSD 8.68% for tissue homogenates. Typical linear regression equations, correlation coefficients in plasma and each tissue were listed in Table 1.

#### 3.1.3. Precision and Accuracy

The intraday and interday precision and accuracy were assessed by analyzing six aliquots of low, medium, and high concentration samples. The intraday precision of (4*E*,6*E*,12*E*)-tetradecatriene-8,10-diyne-1,3-diyl diacetate in rat plasma and tissues ranged between 1.83 and 6.71% with RE of −14.47~−7.81% and the interday precision ranged between 1.57 and 8.68% with RE of −14.38~−5.13%.

#### 3.1.4. Recovery and Stability

The extraction recoveries of (4*E*,6*E*,12*E*)-tetradecatriene-8,10-diyne-1,3-diyl diacetate ranged from 86.6% to 91.6% in plasma and tissue samples and the method recovery ranged from 85.6% to 94.9%, while the recovery of IS was above 80%. These data indicated that the biosample preparation procedure was satisfied and can achieve the acceptable extraction recovery. Stability of analytes showed no significant sample loss over 12 h at room temperature, three freeze-thaw cycles, and 10 days storage condition. The RE of three conditions were within ±15%.

### 3.2. Pharmacokinetics of (4*E*,6*E*,12*E*)-Tetradecatriene-8,10-diyne-1,3-diyl Diacetate in Rats

**Figure 3:**
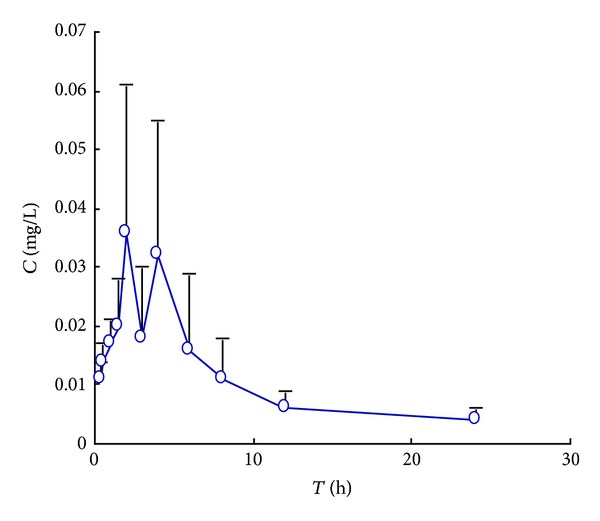
Mean plasma concentration-time curve of (4*E*,6*E*,12*E*)-tetradecatriene-8,10-diyne-1,3-diyl diacetate after oral administration of raw *Atractylodis Rhizoma* (40 g·kg^−1^) to rats. (mean ± SD, *n* = 6).

**Figure 4:**
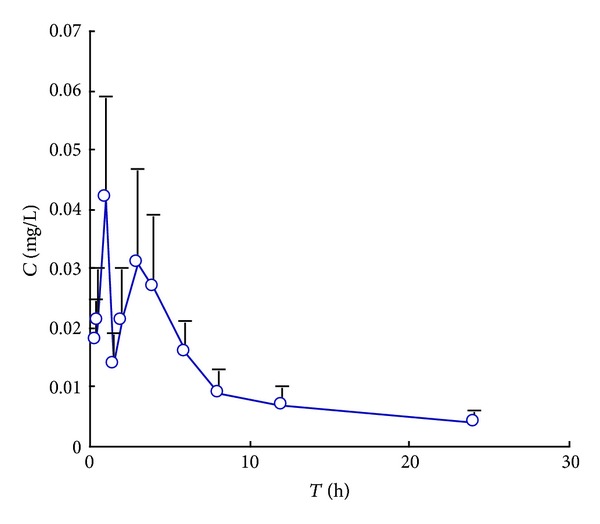
Mean plasma concentration-time curve of (4*E*,6*E*,12*E*)-tetradecatriene-8,10-diyne-1,3-diyl diacetate after oral administration of processed *Atractylodis Rhizoma* (40 g·kg^−1^) to rats. (mean ± SD, *n* = 6).

The assay was applied to a preliminary pharmacokinetic experiment after oral administration of 40 g·kg^−1^ raw and processed *Atractylodis Rhizoma* to rats, respectively. Mean concentration-time curves were shown in Figures 3 and 4. The pharmacokinetic parameters were shown in Table 2.

A significant result of this study is finding that (4*E*,6*E*,12*E*)-tetradecatriene-8,10-diyne-1,3-diyl diacetate showed double peaks after oral administration, which demonstrated that a hepatoenteral circulation may exist. For raw *Atractylodis Rhizoma* the absorption peaks in rat plasma was at 2 h and 4 h, respectively and the *C*_max⁡_ was 38 ± 24 *μ*g·L^−1^. And for processed *Atractylodis Rhizoma* the absorption peaks was at 1 h and 3 h, respectively and the *C*_max⁡_ is 42 ± 17 *μ*·gL^−1^. So the time of absorption peak was 1 hour in advance and the concentration of rat plasma was increased after processing. The value of *T*_max⁡_ and *T*_1/2_ indicated that the (4*E*,6*E*,12*E*)-tetradecatriene-8,10-diyne-1,3-diyl diacetate was rapidly distributed but slowly eliminated. The reason for this result also requires further study.

### 3.3. Tissue Distribution Study

**Figure 5:**
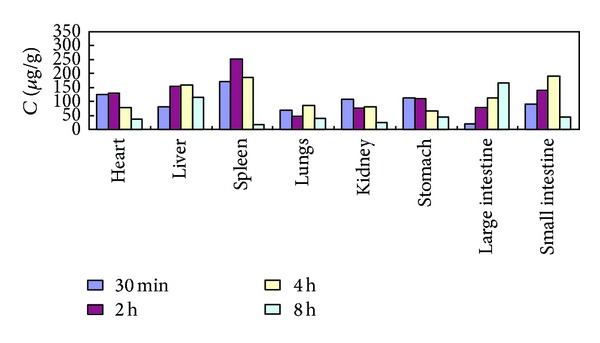
The concentration-time profile of (4*E*,6*E*,12*E*)-tetradecatriene-8,10-diyne-1,3-diyl diacetate in tissues after oral administration of raw* Atractylodis Rhizoma* at a dose of 40 g·kg^−1^ to rats (*n* = 6).

**Figure 6:**
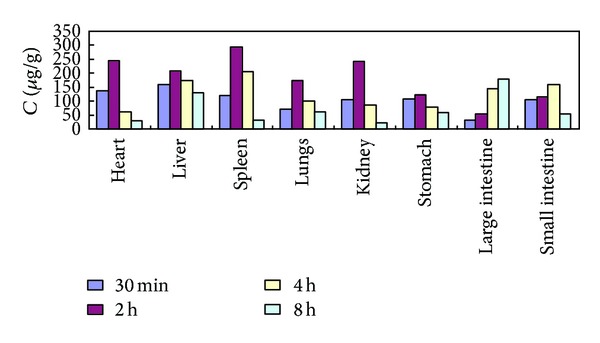
The concentration-time profile of (4*E*,6*E*,12*E*)-tetradecatriene-8,10-diyne-1,3-diyl diacetate in tissues after oral administration of processed *Atractylodis Rhizoma* at a dose of 40 g·kg^−1^ to rats (*n* = 6).

The tissue concentrations of (4*E*,6*E*,12*E*)-tetradecatriene-8,10-diyne-1,3-diyl diacetate determined at 0.5, 2, 4, and 8 h after oral administration raw and processed *Atractylodis Rhizoma*at a dose of 40 g·kg^−1^are shown in Table 3 and Figures 5 and 6, which indicated that (4*E*,6*E*,12*E*)-tetradecatriene-8,10-diyne-1,3-diyl diacetate could be distributed to all collected tissues, for raw *Atractylodis Rhizoma, *the concentrations of (4*E*,6*E*,12*E*)-tetradecatriene-8,10-diyne-1,3-diyl diacetate in tissue were distributed, followed by spleen, liver, small intestine, heart, stomach, large intestine, kidney, and lungs; for processed *Atractylodis Rhizoma, *the concentrations of (4*E*,6*E*,12*E*)-tetradecatriene-8,10-diyne-1,3-diyl diacetate in tissue were distributed, followed by spleen, heart, kidney, liver, lungs, stomach, small intestine, large intestine. Relatively, the concentrations of (4*E*,6*E*,12*E*)-tetradecatriene-8,10-diyne-1,3-diyl diacetate of raw and processed *Atractylodis Rhizoma *was higher in the spleen. And the concentrations in the spleen were increased after stir-frying with bran. The results showed that processing can promote the rate of absorption of (4*E*,6*E*,12*E*)-tetradecatriene-8,10-diyne-1,3-diyl diacetate.

## 4. Conclusion

A simple, specific, and rapid RP-HPLC method with UV detection for quantification of (4*E*,6*E*,12*E*)-tetradecatriene-8,10-diyne-1,3-diyl diacetate in rat plasma has been developed for the first time. It has been successfully applied to a preliminary pharmacokinetic and tissue distribution study of (4*E*,6*E*,12*E*)-tetradedecatriene-8,10-diyne-1,3-diyl diacetate after oral administration of 40 g·kg^−1^ raw and processed *Atractylodis Rhizoma,* respectively. We found that the *T*_max⁡_ have significant difference (*P* < 0.05), and other pharmacokinetics have no significant difference after using Student's* t-*test. The result indicated that processing can promote and accelerate the absorption and the concentration of (4*E*,6*E*,12*E*)-tetradecatrinen-8,10-diyne-1,3-diyl diacetate is the highest in the spleen Which proved that the traditional theory of processing *Atractylodis Rhizoma* can increase its function of tonifying the spleen.

## Figures and Tables

**Table 1:** The Linear regression analysis of (4*E*,6*E*,12*E*)-tetradecatriene-8,10-diyne-1,3-diyl diacetate in rat plasma and tissues (*n* = 7).

Sample	Calibration curves	*r*	Linear range
Plasma	*Y* = 23.628*X* + 0.044	0.9950	0.00361–0.18814
Heart	*Y* = 13.259*X* + 0.031	0.9958	0.00149–0.07322
Liver	*Y* = 14.351*X* + 0.064	0.9951	0.00144–0.06925
Spleen	*Y* = 12.936*X* + 0.028	0.9951	0.00146–0.07431
Lungs	*Y* = 14.704*X* + 0.059	0.9956	0.00143–0.07872
Kidney	*Y* = 12.936*X* + 0.028	0.9951	0.00143–0.06716
Stomach	*Y* = 15.161*X* + 0.012	0.9964	0.00143–0.06786
Large intestine	*Y* = 11.271*X* + 0.019	0.9952	0.00146–0.08179
Small intestine	*Y* = 12.956*X* + 0.037	0.9985	0.00144–0.07339

**Table 2:** The pharmacokinetic parameters of (4*E*,6*E*,12*E*)-tetradecatriene-8,10-diyne-1,3-diyl diacetate of raw and processed *AtractylodisRhizoma* at a dose of 40 g·kg^−1^ to rats, respectively (*n* = 6).

Parameters	Raw	Processed
AUC_(0-*t*)_ (mg·h·L^−1^)	0.260 ± 0.137	0.265 ± 0.100
AUC_(0-*∞*)_ (mg·h·L^−1^)	0.317 ± 0.157	0.303 ± 0.111
MRT_(0-*t*)_ (h )	7.706 ± 0.888	7.384 ± 0.86
MRT_(0-*t*)_ (h)	14.86 ± 6.813	12.22 ± 3.364
*t*_1/2*z*_ (h)	9.905 ± 7.382	8.047 ± 3.347
*T*_max⁡_ (h)	2.333 ± 0.816	1.000 ± 0.817
*C*_max⁡_ (mg·L^−1^)	0.038 ± 0.024	0.042 ± 0.017

**Table 3:** The tissue concentrations of (4*E*,6*E*,12*E*)-tetradecatriene-8,10-diyne-1,3-diyl diacetate after oral administration raw and processed *Atractylodis Rhizoma* at a dose of 40 g·kg^−1^ to rats, respectively (*μ*g/g).

Tissues	0.5 h		2 h		4 h		8 h	
	Raw	Processed	Raw	Processed	Raw	Processed	Raw	Processed
Heart	125.31	137.68	128.97	244.30	77.35	61.62	35.94	30.56
Liver	80.37	158.37	155.10	208.56	158.07	173.38	115.10	128.98
Spleen	170.71	119.41	253.18	292.69	186.45	204.92	17.34	30.66
Lung	69.35	70.01	46.54	174.26	84.79	100.26	40.20	60.61
Kidney	108.76	105.54	76.15	242.21	81.83	84.78	24.51	21.12
Stomach	112.31	107.95	109.77	122.96	65.22	78.53	43.17	59.93
Large Intestine	20.33	30.68	79.02	54.78	112.25	143.34	166.77	176.64
Small Intestine	91.25	106.29	139.04	115.53	191.22	159.02	44.91	54.78
